# 成人急性白血病自体造血干细胞移植中国专家共识（2024年版）

**DOI:** 10.3760/cma.j.cn121090-20240611-00218

**Published:** 2024-07

**Authors:** 中华医学会血液学分会造血干细胞应用学组

## Abstract

自体造血干细胞移植（autologous stem cell transplantation，ASCT）是成人急性白血病（AL）缓解后治疗的方法之一。与异基因造血干细胞移植（allo-HSCT）相比，ASCT具有不受供者限制、不发生移植物抗宿主病（GVHD）、移植相关死亡率低等优点；与多疗程的巩固化疗相比，ASCT能显著缩短治疗时间、减轻患者经济负担，提升患者生活质量。但目前，我国成人AL患者接受ASCT数量较少。为提高临床医师对ASCT在成人AL治疗中作用和地位的认识，提高ASCT临床疗效，亟待制定我国成人急性白血病自体造血干细胞移植专家共识。

## Abstract (en)

Autologous stem cell transplantation（ASCT）emerges as a therapeutic strategy following remission in adult acute leukemia（AL）. It offers advantages over allogeneic hematopoietic stem cell transplantation（allo-HSCT）, including independence from donor availability, absence of graft-versus-host disease（GVHD）, and a reduced risk of transplant-related mortality. Furthermore, when juxtaposed with the extended regimens of consolidation chemotherapy, ASCT stands out by markedly abbreviating treatment duration, alleviating the economic strain on patients, and enhancing their overall quality of life. Despite these benefits, the adoption of ASCT among adult AL patients in China remains disproportionately low. To enhance clinical physicians' understanding of the role and position of ASCT in AL management and to improve the clinical efficacy of ASCT, it is urgent to establish a consensus among experts on ASCT for adult acute leukemia in our nation.

自体造血干细胞移植（autologous hematopoietic stem cell transplantation, auto-HSCT）是成人急性白血病（acute leukemia, AL）缓解后的治疗方法之一。与异基因造血干细胞移植（allogeneic hematopoietic stem cell transplantation, allo-HSCT）相比，auto-HSCT具有不受供者限制、不发生移植物抗宿主病（graft versus host disease, GVHD）、移植相关死亡率低等优点；与多疗程的巩固化疗相比，auto-HSCT能显著缩短治疗时间、减轻患者经济负担，提升患者生活质量[1]–[5]。但目前，我国成人AL患者接受auto-HSCT数量较少。为提高临床医师对auto-HSCT在成人AL治疗中作用和地位的认识、提高auto-HSCT临床疗效，中华医学会血液学分会造血干细胞应用学组制定并发布本共识。

一、auto-HSCT治疗成人AL的适应症

auto-HSCT适用与否取决于患者对治疗的耐受性、疾病危险度分层、移植前疾病状态及有无合适供者等因素。适合移植的AL患者需全程监测微小残留病/可检测残留病（MRD）状态，MRD持续阴性[6]–[7]（连续检测至少两次以上）患者方可行auto-HSCT。MRD检测手段主要包括多参数流式细胞术（MFC）、实时定量聚合酶链反应（RQ-PCR）技术、数字PCR（dd-PCR）及二代测序（NGS）技术等高通量检测技术，其中dd-PCR和NGS尚处于临床研究阶段，未被常规用于MRD评估[8]–[9]。高龄（≥65岁）并非auto-HSCT的绝对禁忌[1]，除年龄外，还应考虑患者体能状况及心、肺、肝、肾等脏器功能，建议结合造血干细胞移植合并症指数（HCT-CI）等特定风险评估模型进行综合评估。

（一）急性髓系白血病（AML）

auto-HSCT的成人AML适应证包括：①预后良好/中等组，1个疗程诱导化疗获得第1次完全缓解（CR1）且MRD持续阴性患者[2],[10]–[11]；②预后良好/中等组，2个疗程化疗获得CR1且MRD持续阴性患者[2]–[4],[11]；③首次复发后达第2次CR（CR2）且PML-RARα融合基因阴性的急性早幼粒细胞白血病（APL）患者[12]–[14]。详见图1。

**图1:**
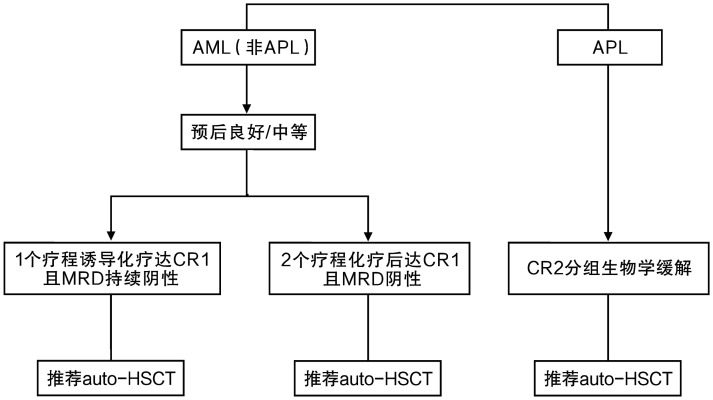
成人急性髓系白血病（AML）患者自体造血干细胞移植（auto-HSCT）的适应证选择[2]–[4],[10]–[14] **注** APL：急性早幼粒细胞白血病；CR1、CR2：第1、2次完全缓解；MRD：微小残留病

（二）急性淋巴细胞白血病（ALL）

auto-HSCT的ALL适应证包括：①预后良好组费城染色体阴性（Ph^−^）获得CR1、MRD持续阴性且充分巩固强化治疗后的患者[9],[15]；②预后不良组Ph^−^、MRD持续阴性且无合适供者或不适合allo-HSCT的CR1患者[15]–[16]；③治疗3个月内标准化疗后实现完全分子学缓解并持续至移植的费城染色体阳性（Ph^+^）患者[16]–[18]。详见图2。

**图2:**
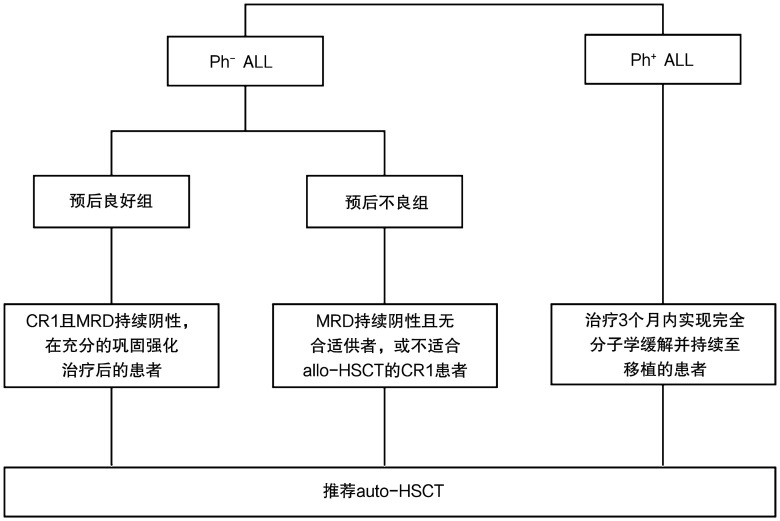
成人急性淋巴细胞白血病（ALL）自体造血干细胞移植（auto-HSCT）的适应证选择[9],[15]–[18] **注** Ph：费城染色体；CR1：第1次完全缓解；MRD：微小残留病

二、移植时机的选择

（一）AML

建议适合的AML患者获得CR后，接受1～3个疗程的巩固治疗[2]–[5]，其中含有至少1个疗程中/大剂量Ara-C为基础的方案[2]–[5]，并在巩固治疗期间完成外周血干细胞动员及采集，随后实施auto-HSCT。

（二）ALL

建议适合的ALL患者获得CR后接受1～2个疗程巩固强化治疗，实施造血干细胞动员及采集，随后完成3～4个疗程的巩固治疗（应含有大剂量MTX方案），实施auto-HSCT[19]。Ph^+^ ALL的巩固化疗应保证酪氨酸激酶抑制剂（TKI）的用药。预防中枢神经系统白血病（CNSL）在ALL治疗中尤为重要，ALL患者在auto-HSCT前应至少进行6次鞘内注射以预防CNSL发生[19]–[20]。

接受auto-HSCT的AL患者，在诱导化疗和巩固治疗全程监测患者MRD状态，在确认患者的骨髓及采集物均为MRD阴性后，方可进行auto-HSCT。

三、自体造血干细胞动员、采集与保存

造血干细胞主要来源于外周血和骨髓。自体外周血干细胞具有易采集、易于冷冻保存、移植后造血重建较快、移植相关死亡率较低等优势[19],[21]，在临床广泛得到应用。

（一）外周血干细胞动员

动员策略包括化疗联合细胞因子动员和稳态细胞因子动员。

推荐化疗联合细胞因子的动员方式，因其具有抗肿瘤、提高采集干细胞数量进而减少采集次数等优势[22]。

化疗联合细胞因子动员策略：自化疗后7～10 d（最迟为白细胞最低点时）始，予粒细胞集落刺激因子（G-CSF）5～10 µg·kg^−1^·d^−1^，持续至采集结束。

患者外周血白细胞、CD34^+^细胞数通常在予G-CSF后4～6 d到达高峰[23]，由于骨髓抑制程度不同，CD34^+^细胞达到高峰的时间可有一定波动[24]。推荐在造血恢复期每日监测外周血白细胞和CD34^+^细胞计数，白细胞计数恢复至>5×10^9^/L或外周血CD34^+^细胞>0.02×10^9^/L时采集外周造血干细胞[22],[25]–[27]。

当患者外周血CD34^+^细胞计数（0.015～0.02）×10^9^/L时，建议开始采集[22],[24],[27]。若3～4次采集不能获得目标CD34^+^细胞数量应考虑待患者血象恢复后行稳态细胞因子动员（G-CSF 10 µg·kg^−1^·d^−1^）或更换化疗方案动员[22]。

（二）自体造血干细胞和骨髓采集

1. 自体外周血干细胞采集：利用血细胞分离机采集单个核细胞层，获得动员的外周血干细胞（PBSC）。CD34^+^细胞最低输注量为2×10^6^/kg[27]–[28]。尽管更高的CD34^+^细胞输注量与更快的植入有关，但过高的CD34^+^细胞输注量（>7×10^6^/kg）可能增加疾病复发风险[29]。

2. 自体骨髓采集：自体骨髓移植时，所采集骨髓有核细胞数应达（1～3）×10^8^/kg体重。如需进行体外净化，则骨髓有核细胞数需≥3×10^8^/kg[30]。外周血采集CD34^+^细胞总量不足（连续2 d CD34^+^细胞计数<1×10^6^/kg）时也可采集骨髓作为补充。通常在全身麻醉或局部区域麻醉下进行，选取髂骨后嵴多点穿刺采集骨髓，骨髓的安全采集量不超过20 ml/kg。

（三）自体造血干细胞保存

auto-HSCT患者从造血干细胞采集到输注历时数日至数月不等，选择恰当的冻存方式以保持造血干细胞活性尤为重要。通过程控降温在液氮冻存和（或）深低温冰箱冻存是长期保存造血干细胞的有效方法[31]。复苏后干细胞的质控目前尚无统一标准，有条件的单位可开展CD34^+^细胞的活率检测[32]–[33]。

四、预处理方案及造血干细胞输注

（一）预处理方案

auto-HSCT预处理方案主要分为全身放射治疗（TBI）方案和非TBI方案两种，常用预处理方案见表1。

**表1:** 成人急性髓系白血病（AML）和急性淋巴细胞白血病（ALL）患者自体造血干细胞移植（auto-HSCT）预处理方案推荐

预处理方案	药物及剂量	适用疾病
Bu/Cy	Bu 3.2 mg·kg^−1^·d^−1^×4 d；Cy 60 mg·kg^−1^·d^−1^×2 d	AML[41]–[42]
Bu/Mel	Bu 3.2 mg·kg^−1^·d^−1^×3～4 d；Mel 140 mg/m^2^×1 d（>60岁者减量为120 mg/m^2^）	AML[34],[38],[43]
Bu/Cy/Flu/Ara-C	Bu 3.2 mg·kg^−1^·d^−1^×3～4 d；Cy 40～50 mg·kg^−1^·d^−1^×2 d；Flu 30 mg·kg^−1^·d^−1^×3 d；Ara-C 1～2 g·m^−2^·d^−1^×3 d	AML[36]
Bu/Cy/CLAD/Ara-C	Bu 3.2 mg·kg^−1^·d^−1^×3 d；Cy 40 mg·kg^−1^·d^−1^×2 d；CLAD 10 mg/d×3 d；Ara-C 2 g·m^−2^·d^−1^×3 d	AML[37]
TBI/Cy	TBI 3.3 Gy，−9～−7 d；Cy 40 mg·kg^−1^·d^−1^，−6、−5 d	ALL[44]
TBI/Cy/Flu/Ara-C	TBI 7 Gy，−7 d或3.3 Gy，−9～−7 d；Cy 40 mg·kg^−1^·d^−1^，−6、−5 d；Flu 30 mg·m^−2^·d^−1^，−4～−2 d；Ara-C 2 g·m^−2^·d^−1^，−4～−2 d	ALL[16]–[18]
Bu/Cy/Flu/Ara-C	Bu 3.2 mg·kg^−1^·d^−1^，−9～−7 d；Cy 40 mg·kg^−1^·d^−1^，−6、−5 d；Flu 30 mg·m^−2^·d^−1^，−4～−2 d；Ara-C 2 g·m^−2^·d^−1^，−4～−2 d	ALL[17]–[18]

**注** Bu：白消安；Cy：环磷酰胺；Mel：美法仑；Flu：氟达拉滨；Ara-C：阿糖胞苷；CLAD：克拉屈滨；TBI：全身放射治疗

1. AML：①白消安（Bu）/环磷酰胺（Cy）方案是AML患者auto-HSCT最常用的预处理方案，有较强的免疫抑制性[2],[5],[10],[22],[34]–[35]。②Bu/Cy/氟达拉滨（Flu）/阿糖胞苷（Ara-C）[4],[36]、Bu/Cy/克拉屈滨（CLAD）/Ara-C[37]等改良预处理方案加强了预处理的抗肿瘤作用，是降低移植后复发的有效方案。③白消安/美法仑（Bu/Mel）方案也是可供AML患者选择的一种预处理方案，在低危组患者中更具优势[34],[38]。

2. ALL：①TBI/Cy方案是ALL患者auto-HSCT的常用预处理方案[22],[39]。②Flu+Ara-C联合Bu/Cy或TBI+Cy也是安全有效的预处理方案[16],[40]。

（二）自体造血干细胞输注

输注前需解冻复苏，该过程会影响细胞活性。冻存细胞经37 °C～42 °C水浴箱在1 min内迅速解冻，复苏后应尽快开始输注外周血干细胞，若患者病情允许，每袋细胞应在20 min内快速输注，以减少二甲基亚砜（DMSO）对造血干细胞的损害。建议每次输注干细胞量不超过500 ml（即DMSO小于50 ml），如果冻存细胞体积过大，可以分日输注。同时注意监测患者生命体征及尿色、尿量等[22]。

五、自体造血干细胞移植后造血与免疫重建

（一）造血重建的监测

APBSCT一般在移植后2周造血重建（植入），较自体骨髓移植（ABMT）快，移植后造血重建的时间受回输物中CD34^+^细胞数量、是否液氮冻存、DMSO浓度等多种因素影响[45]。若患者移植后28 d，存在两系或三系细胞计数降低（中性粒细胞计数≤0.5×10^9^/L，血小板计数≤20×10^9^/L，血红蛋白浓度≤70 g/L）持续3 d以上，则提示植入不良[22]，可能与患者的造血干细胞和骨髓微环境有关，临床可应用间充质干细胞、G-CSF、重组人血小板生成素（rhTPO）、血小板生成素受体激动剂（TPO-RA）等促进造血重建。

（二）免疫重建的监测

auto-HSCT后免疫重建较缓慢（至少需要数月）[46]，处于免疫缺陷状态下的患者易发生感染[47]，建议有条件的单位对auto-HSCT后患者进行免疫重建监测（包括T细胞、B细胞和NK细胞亚群等）[22],[48]。

六、移植后维持治疗

移植后原发病复发是auto-HSCT失败的主要原因。在患者造血重建后，应尽早开始维持治疗。

（一）AML

有研究显示auto-HSCT后应用白细胞介素-2（IL-2）皮下注射维持治疗可能是改善中、低危AML患者预后的有效方法，部分患者可能受益[49]。在AML患者中，auto-HSCT后的维持治疗作用仍然不明确，尚需前瞻性临床试验进一步验证。

（二）ALL

目前，auto-HSCT后的维持治疗主要应用于ALL患者，维持治疗方案通常包括维持化疗和免疫治疗等[20],[50]–[51]。

1. 维持化疗：当患者移植后白细胞计数恢复至3×10^9^/L且血小板计数达50×10^9^/L以上时开始接受维持治疗，推荐VP方案和MM方案交替应用，共1～1.5年[16],[20],[50]。

2. 酪氨酸激酶抑制剂（TKI）：推荐Ph^+^ ALL患者auto-HSCT后在VP方案或MM方案的维持治疗基础上联合TKI维持治疗，至少1年[16]–[18]。

3. 免疫治疗：难治/复发急性B淋巴细胞白血病（B-ALL）患者应用CD3/CD19双特异性T细胞衔接器免疫治疗药物和靶向CD22抗原的抗体偶联药物可提高CR率及MRD阴性率[52]–[53]。也有报道难治/复发B-ALL患者行allo-HSCT后应用CAR-T细胞治疗可提高CR率及MRD转阴率[54]–[55]。有条件的单位可探索B-ALL患者auto-HSCT后行CAR-T细胞免疫治疗方案。也有CD3/CD19双特异性T细胞衔接器免疫治疗用于allo-HSCT后维持治疗的报道[56]。有条件的单位可针对高危B-ALL患者探索auto-HSCT后应用靶向CD19或CD22等免疫治疗药物的维持治疗方案，以降低auto-HSCT后的复发率。

七、移植后疗效监测及管理

（一）移植后复发监测

auto-HSCT应全程监测疾病状态，MRD监测尤为重要。建议移植后行骨髓细胞形态学、细胞遗传学检测、骨髓和（或）外周血MRD检测，必要时可行PET-CT检查。推荐监测频率：auto-HSCT后3个月内每月复查1次，3个月至2年每3个月复查1次，2～3年每6个月复查1次，3～5年每年复查1次，同时进行相应指标的检测并根据患者病情变化及时调整治疗。对于结合临床表现怀疑病情变化的患者，任意时间点均推荐进行上述检测；若检测结果不稳定，则需缩短本次至下次监测的间隔。

（二）移植后患者的随访及管理

所有auto-HSCT后的患者均应进行定期随访，以了解患者病情变化、及时进行康复指导。随访内容建议包括：①骨髓细胞形态学和MRD的监测；②体格检查、血常规和主要脏器（如心、肝、肺、肾等）功能的实验室检查、肿瘤标志物检测和影像学检查；③注意监测移植后有无肝炎病毒、巨细胞病毒等多种病毒复制；④接受TBI的患者还应关注晶状体、垂体功能、甲状腺功能等；⑤建议有条件的移植单位可对auto-HSCT后患者生活质量进行随访，并根据患者生活质量状况提供必要建议。

八、结语

auto-HSCT是成人AL的一种重要的治疗方法，在临床实践中应严格把握适应症，注意移植前足量化疗的重要性，密切监测MRD水平，及时、足量采集自体造血干细胞，选择适合的预处理方案并重视移植后维持治疗。本共识将有助于提高我国auto-HSCT治疗成人AL的临床疗效，进而改善成人AL患者的auto-HSCT预后。
